# Catheter-associated urinary tract infections (CAUTIs) and non-CAUTI hospital-onset urinary tract infections: Relative burden, cost, outcomes and related hospital-onset bacteremia and fungemia infections

**DOI:** 10.1017/ice.2024.26

**Published:** 2024-07

**Authors:** Timothy Kelly, ChinEn Ai, Molly Jung, Kalvin Yu

**Affiliations:** Department of Medical Affairs, Becton, Dickinson and Company, Franklin Lakes, NJ, USA

## Abstract

**Objective:**

To describe the relative burden of catheter-associated urinary tract infections (CAUTIs) and non-CAUTI hospital-onset urinary tract infections (HOUTIs).

**Methods:**

A retrospective observational study of patients from 43 acute-care hospitals was conducted. CAUTI cases were defined as those reported to the National Healthcare Safety Network. Non-CAUTI HOUTI was defined as a positive, non-contaminated, non-commensal culture collected on day 3 or later. All HOUTIs were required to have a new antimicrobial prescribed within 2 days of the first positive urine culture. Outcomes included secondary hospital-onset bacteremia and fungemia (HOB), total hospital costs, length of stay (LOS), readmission risk, and mortality.

**Results:**

Of 549,433 admissions, 434 CAUTIs and 3,177 non-CAUTI HOUTIs were observed. The overall rate of HOB likely secondary to HOUTI was 3.7%. Total numbers of secondary HOB were higher in non-CAUTI HOUTIs compared to CAUTI (101 vs 34). HOB secondary to non-CAUTI HOUTI was more likely to originate outside the ICU compared to CAUTI (69.3% vs 44.1%). CAUTI was associated with adjusted incremental total hospital cost and LOS of $9,807 (*P* < .0001) and 3.01 days (*P* < .0001) while non-CAUTI HOUTI was associated with adjusted incremental total hospital cost and LOS of $6,874 (*P* < .0001) and 2.97 days (*P* < .0001).

**Conclusion:**

CAUTI and non-CAUTI HOUTI were associated with deleterious outcomes. Non-CAUTI HOUTI occurred more often and was associated with a higher facility aggregate volume of HOB than CAUTI. Patients at risk for UTIs in the hospital represent a vulnerable population who may benefit from surveillance and prevention efforts, particularly in the non-ICU setting.

## Introduction

Urinary tract infections (UTIs) were the most common healthcare-associated infection (HAI) in 2002 accounting for 36% of all HAIs.^1^ However, the HAI landscape has changed substantially—a 2015 point-prevalence analysis found catheter-associated urinary tract infections (CAUTIs) to be the fifth most common HAI.^2^ The decline in hospital-onset UTIs (HOUTIs) may be attributed in part to preventability of CAUTI^3^ and the availability and adoption of guidelines for mitigating those infections.^4^

Changes to the definition of CAUTI in 2009 and 2015 also likely contributed to the decline in prevalence. The first definition modification removed asymptomatic bacteriuria,^5^ and the second excluded both urine cultures that were positive for non-bacterial pathogens and those with colony counts below 100,000 colony-forming units per milliliter (CFU/mL).^6^ The CAUTI definition updates appear primarily responsible for the decline in CAUTI rates,^7^ while not seeming to impact positive urine cultures rates.^8^

The definition for CAUTI is stringent so it is likely that the majority of HOUTIs are non-CAUTI.^9^ With pay-for-performance metrics focused on CAUTIs, these non-CAUTI HOUTIs have not been studied extensively. Prior research has shown a definitional change in CAUTI can increase the cases of reportable central line-associated bloodstream infection (CLABSI) events.^10^ Understanding how non-CAUTI HOUTIs are associated with secondary bloodstream infections may be important in light of the new, proposed HAI metric for hospital-onset bacteremia and fungemia (HOB) that is under development.^11^

This analysis divides HOUTIs into two groups: 1) CAUTIs and 2) non-CAUTI HOUTIs and characterizes the clinical and economic burden of each group, examining their impact upon secondary HOB, total hospital costs, and length of stay (LOS).

## Methods

### Study design and population

This was a retrospective, observational study that utilized data from the Becton, Dickinson and Company (BD) Insights Research Database (BD, Franklin Lakes, New Jersey) from October 2015 to June 2019, which includes electronically captured laboratory, pharmacy, patient demographic, admission, discharge, and transfer data. Details of the data collection system have been previously described.^12,13^

The facility-level aggregate burden of HOUTIs, and association between HOUTIs and secondary HOB, were estimated in adults aged 18 years or older with hospital LOS between 2 and 365 days without other HAIs (Cohort 1). Financial data, including actual cost of care for an admission, were available on a subset of hospitals so a second cohort was constructed. The attributable hospital cost and LOS of CAUTI and non-CAUTI HOUTIs were estimated in a nested sample (Cohort 2). Recognizing that patients who get CAUTI or non-CAUTI HOUTIs are different, several additional inclusion criteria were applied to improve comparability. The control group was comprised of patients meeting the following criteria: (1) LOS ≥2 days; (2) no antimicrobial therapy ≥72 hours following admission; (3) no diagnosis-related group (DRG) for infection; and (4) no other positive culture collected during the hospitalization period.^14,15^ Patients with missing intensive care unit (ICU) status and those where the age distribution, major diagnostic category (MDC), DRG, or primary procedure were different from patients with CAUTI were excluded.

### Exposure measures

Patients with HOUTI were defined using an algorithm which included: (1) presentation with a positive urine culture; (2) containing ≤2 non-commensal isolates (>10,000 colony-forming units/ml), on day 3 or later (ie, hospital-onset period); and (3) likely antimicrobial susceptibility testing (AST) predicated on the mechanism of specimen collection and identified pathogen consistent with the American Society of Microbiology’s (ASM) Clinical Microbiology Procedures Handbook.^16^ In addition to a positive urine culture of a likely hospital-onset infection, all HOUTIs in this analysis were required to have a second qualification in order to maximize likelihood of clinical significance: a new antimicrobial, appropriate for managing the uropathogen, prescribed within 2 days of the collection date of the first positive urine culture. The time frame of the new antimicrobial use is consistent with the Centers for Disease Control and Prevention (CDC) Sepsis Surveillance Toolkit^17^ and defined elsewhere.^11^ CAUTI was defined using the National Healthcare Safety Network (NHSN) definition and was confirmed by hospital infection preventionists.^18^ Non-CAUTI HOUTIs were defined as those HOUTIs not meeting the CAUTI definition (Table 1).

**Table 1.** Prevalence analysis of all cases of HOUTI (Cohort 1)

Prevalence	CAUTI (Reported to NHSN)	Non-CAUTI HOUTI
	CAUTI	Non-CAUTI HOUTI
	***N*** **= 463**	***N*** **= 4,730**
Excluded (no antimicrobials, or no appropriate antimicrobials ordered)	29 (6.3%)	1,553 (32.8%)
Cases (cases with new antimicrobial order appropriate for the urinary pathogen)	434 (93.7%)	3,177 (67.2%)
**Hospital-onset bacteremia and fungemia (HOB)**	**CAUTI**	**Non-CAUTI HOUTI**
	***N*** **= 434**	***N*** **= 3,177**
No HOB	379 (87.3%)	3,008 (94.7%)
HOB with a pathogen different from the urine pathogen	21 (4.8%)	68 (2.1%)
HOB with a matched urinary pathogen	34 (7.8%)	101 (3.2%)
**Urine specimen collection location—All**	**CAUTI**	**Non-CAUTI HOUTI**
	***N*** **= 434**	***N*** **= 3,177**
ICU	231 (53.2%)	690 (21.7%)
Non-ICU	203 (46.8%)	2,487 (78.3%
**Urine specimen collection location—secondary HOB**	**CAUTI**	**Non-CAUTI HOUTI**
	***N*** **= 34**	***N*** **= 101**
ICU	19 (55.9%)	31 (30.7%)
Non-ICU	15 (44.1%)	70 (69.3%)

### Outcome measures

Potential secondary HOB was defined as a non-contaminated, positive blood culture with a noncommensal HOB/sepsis pathogen, occurring 2 days before or 4 days after the positive urine specimen requiring antimicrobial susceptibility testing and presenting with the same pathogen.^11^ All-cause 30-day readmission, mortality, LOS, and total cost of care to the organization were analyzed.

### Other variables

Age, gender, insurance type, and hospital characteristics were assessed. The ALaRMS score, a severity of illness marker derived from laboratory values which calculates mortality risk during the same hospital admission,^19^ was used to assess the clinical severity of hospitalized patients.

### Statistical analysis

In Cohort 1, the prevalence of CAUTI and non-CAUTI HOUTI were estimated and the association with secondary HOB was evaluated. Patient and hospital characteristics were described overall and by CAUTI, non-CAUTI HOUTI, or control status using frequencies. The risk of secondary HOB, determined by examining the prevalence of HOB in patients with CAUTI and non-CAUTI HOUTI, was quantified. Analyses were stratified by ICU status to understand whether the location of the positive urine collection varied on the risk of HOB.

In Cohort 2, the attributable cost of CAUTI and non-CAUTI HOUTI compared to infection-free controls were estimated using generalized linear mixed models with hospital as random effect to account for within-cluster correlation of data, and adjusted for age, gender, insurance type, ALaRMS severity of illness score, hospital bed-size, teaching status, and urbanicity. For binary outcomes, a binomial regression was used. Poisson regression was used for LOS. Gamma regression was employed for total cost as it was skewed. Since ICU status^20^ and HOB^12^ are known to be associated with higher costs, analyses in Cohort 2 were *a priori* stratified by ICU status (the patient was never admitted to the ICU during their entire LOS—“never,” or the patient spent some portion of their stay in the ICU—“ever”), LOS (≤10 days or >10 days), and potential secondary HOB (yes or no).

All analyses were conducted using R software version 4.1.2 (R Foundation for Statistical Computing, Vienna, Austria) with RStudio (Boston, Massachusetts).

### Ethical considerations

The study was approved as a limited retrospective data set for epidemiological analyses, exempted from consent by the New England Institutional Review Board/WCG and Human Subjects Research Committee (Wellesley, Massachusetts). It was conducted in compliance with Health Insurance Portability and Accountability Act requirements.

## Results

### Prevalence Cohort (Cohort 1)

**Figure 1.**
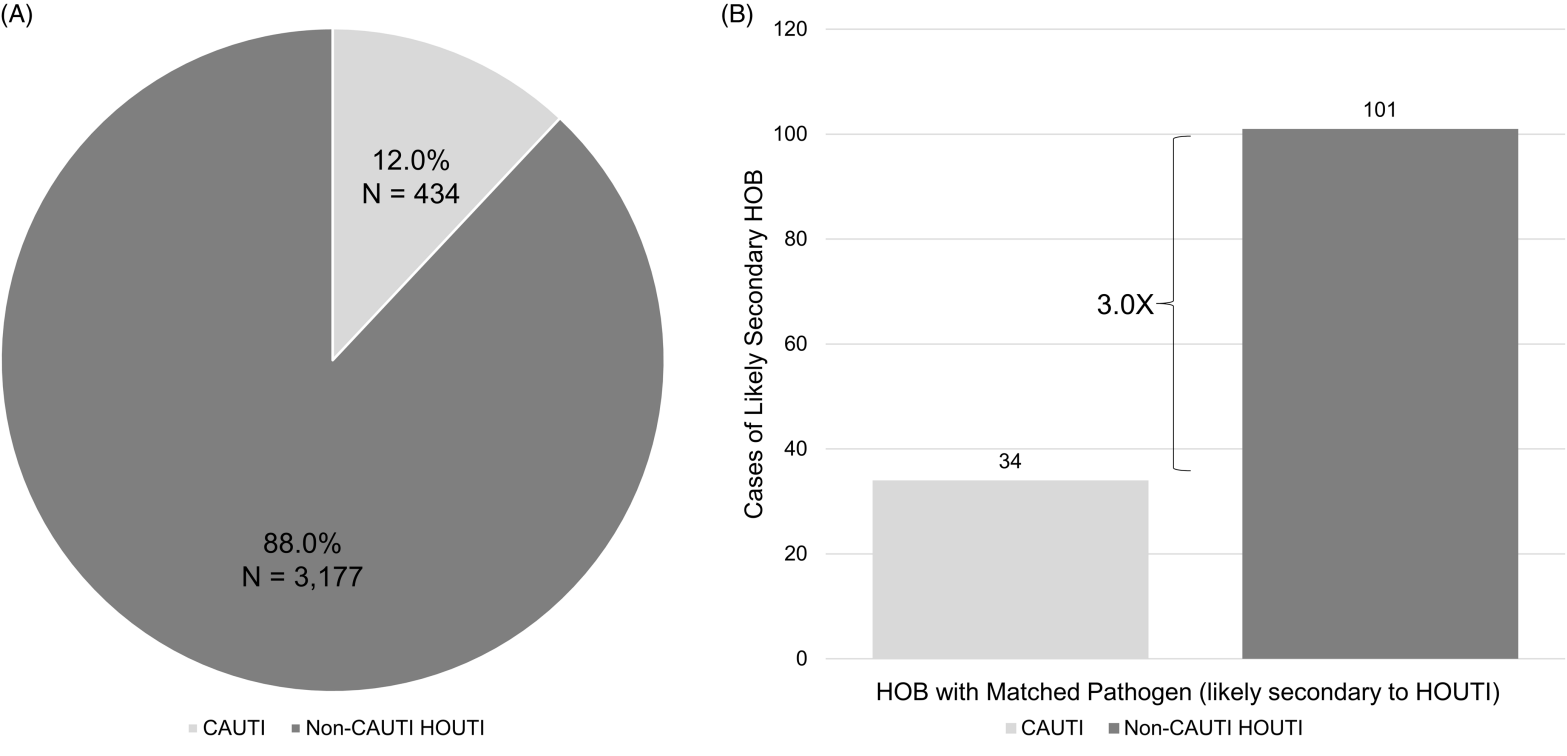
Distribution of HOUTI and attendant risk of secondary HOB. A. Distribution of HOUTI (Prevalence Cohort—Cohort 1). B. Cases of Likely Secondary HOB in Patients with HOUTI (Prevalence Cohort—Cohort 1).

In Cohort 1 of 549,433 hospitalizations (43 hospitals) (Supplemental Table 1), 5,193 patients (0.9%) met the criteria for CAUTI or non-CAUTI HOUTI. Of those 5,193 patients, 463 (8.9%) had CAUTI confirmed by infection preventionists at the facilities. A total of 434 (93.7%) of the CAUTI cases, and 3,177 (67.2%) of the non-CAUTI HOUTI cases, met the criteria for receipt of a new antimicrobial likely targeted at the uropathogen (Table 1; Figure 1A). An analysis by pathogen is presented in Supplemental Table 2.

### Association between CAUTI and non-CAUTI HOUTI with potential secondary HOB

The overall prevalence of likely secondary HOB was 3.7% in patients with CAUTI and non-CAUTI HOUTI. The risk of likely secondary HOB was higher in CAUTI compared to non-CAUTI HOUTI (7.8% vs 3.2%); however, the volume of HOB was threefold greater for non-CAUTI HOUTI compared to CAUTI (101 vs 34, Figure 1B). The location of urine specimen collection was more likely to be in the ICU for CAUTI with secondary HOB compared to non-CAUTI HOUTI with secondary HOB (55.9% vs 30.7%) (Table 1).

### Attributable burden cohort (Cohort 2)

In Cohort 2 of 82,787 hospitalizations (40 hospitals) (Supplemental Table 1), 360 patients (0.4%) had CAUTI and 1,513 (1.8%) had non-CAUTI HOUTI. The mean age was 65.6 years (SD = 15) with the majority of patients being female (52.6%). Patients with CAUTI and non-CAUTI HOUTI were more likely to come from large academic hospitals in urban settings compared to control patients. The ALaRMS score for the same admission mortality risk was highest in CAUTI (M = 56.8, SD = 21.6) followed by non-CAUTI HOUTI (M = 56.4, SD = 19.8) then controls (M = 46.2, SD = 18.8) (Supplemental Table 3).

### Attributable cost of CAUTI and non-CAUTI HOUTI

The attributable risk of increased LOS and total cost were higher in patients with CAUTI and non-CAUTI HOUTI compared to controls, any differential was less clear for readmission and mortality. The overall total adjusted hospital costs for CAUTI patients and their controls were $24,289 (95% CI: $19,861–$29,703) and $14,482 (95% CI: $12,488–$16.794), respectively, yielding an average incremental cost of $9,807 (*P* < .0001). For non-CAUTI HOUTIs and their controls, the overall total adjusted hospital costs were $21,363 (95% CI: $18,124–$25,180) and $14,489 (95% CI: $12,422–$16,899), respectively, yielding an average incremental cost of $6,874 (*P* < .0001) (Table 2).

**Table 2.** Overall findings from the attributable burden Cohort (Cohort 2)

Outcome	Case	Control	Difference/ratio
**CAUTI vs control**			
Readmission	0.07 (0.03, 0.15)	0.09 (0.08, 0.1)	0.82
Mortality	0.08 (0.04, 0.16)	0.02 (0.02, 0.03)	3.78***
Length of stay	7.03 (6.39, 7.73)	4.01 (3.88, 4.16)	3.01***
Total hospital cost	$24,289 (19861, 29703)	$14,482 (12488, 16794)	$9,807***
**Non-CAUTI HOUTI vs control**			
Readmission	0.1 (0.08, 0.14)	0.09 (0.08, 0.1)	1.17
Mortality	0.02 (0.01, 0.04)	0.02 (0.02, 0.03)	1.03
Length of stay	6.98 (6.64, 7.35)	4.01 (3.87, 4.16)	2.97***
Total hospital cost	$21,363 (18124, 25180)	$14,489 (12422, 16899)	$6,874***

Note. *P* value: *<.05; **<.01; ***<.0001. Models were adjusted for age, sex, ALaRMS score, and hospital-level variables (payer, staffed bed size, teaching status, and urbanicity).

Higher attributable cost of CAUTI and non-CAUTI HOUTI compared to controls were generally true in subgroup analyses. However, the magnitude of the association differed. Patients who stayed in the ICU, tended to have worse outcomes. Additionally, the burden of CAUTI and non-CAUTI HOUTI was smallest in LOS ≤10 days with no secondary HOB (Table 3) and much higher in subgroups with LOS >10 days and/or HOB (Supplemental Tables 4 and 5).

**Table 3.** Association between CAUTI and non-CAUTI HOUTI with outcomes in patients with LOS ≤ 10 d and no secondary HOB

	Never-ICU			Ever-ICU		
	CAUTI	Control	Difference/RR	CAUTI	Control	Difference/RR
	***N*** **= 19**	***N*** **= 55,099**		***N*** **= 48**	***N*** **= 20,632**	
**30-d readmission**						
Unadjusted	4 (21.1%)	6231 (11.3%)		3 (6.3%)	2442 (11.8%)	
Adjusted	0.19 (0.07, 0.41)	0.09 (0.08, 0.1)	2.04^ns^	0.05 (0.01, 0.14)	0.09 (0.08, 0.1)	0.53^ns^
**Mortality**						
Unadjusted	0 (0%)	621 (1.1%)		13 (27.1%)	2420 (11.7%)	
Adjusted	–	–	–	0.17 (0.09, 0.3)	0.08 (0.06, 0.1)	2.14*
**Length of stay**						
Unadjusted	7.00 [5.50, 8.00]	3.00 [2.00, 5.00]		8.00 [6.00, 10.0]	4.00 [3.00, 6.00]	
Adjusted	6.93 (5.81, 8.27)	3.86 (3.73, 3.99)	3.07***	7.01 (6.28, 7.82)	4.43 (4.26, 4.6)	2.58***
Unadjusted	12,400 [10,100, 15,400]	11,200 [7,100, 15,600]		24,500 [15,500, 36,700]	14,200 [9,190, 23,400]	
Adjusted	17,903 (13,690, 23,413)	12,464 (10,814, 14,367)	$5,439**	24,041 (18,816, 30,718)	16,704 (13,980, 19,959)	$7,337***

	**Non-CAUTI HOUTI**	**Control**	**Difference/RR**	**Non-CAUTI HOUTI**	**Control**	**Difference/RR**
	**N = 216**	**N = 55,099**		**N = 163**	**N = 20,632**	
**30-d readmission**						
Unadjusted	35 (16.2%)	6231 (11.3%)		16 (9.8%)	2442 (11.8%)	
Adjusted	0.12 (0.09, 0.17)	0.09 (0.08, 0.1)	1.35^ns^	0.07 (0.04, 0.12)	0.09 (0.08, 0.1)	0.81^ns^
**Mortality**						
Unadjusted	2 (0.9%)	621 (1.1%)		21 (12.9%)	2420 (11.7%)	
Adjusted	–	–	–	0.08 (0.05, 0.14)	0.08 (0.06, 0.1)	1.06 ^ns^
**Length of stay**						
Unadjusted	7.00 [6.00, 9.00]	3.00 [2.00, 5.00]		8.00 [6.00, 9.00]	4.00 [3.00, 6.00]	
Adjusted	6.84 (6.44, 7.26)	3.86 (3.73, 3.99)	2.98***	6.88 (6.43, 7.36)	4.42 (4.25, 4.6)	2.46***
**Total hospital cost**						
Unadjusted	14,600 [10,700, 21300]	11,200 [7,100, 15,600]		20,400 [14,100, 29,700]	14,200 [9,190, 23,400]	
Adjusted	18,570 (15,845, 21,763)	12,469 (10,810, 14,383)	$6,101***	22,678 (18,567, 27,699)	16,712 (13,987, 19,967)	$5,966***

Note. *P* value: ^ns^ not statistically significant; *<.05; **<.01; ***<.0001. Models were adjusted for age, sex, ALaRMS score, and hospital-level variables (payer, staffed bed size, teaching status, and urbanicity). *d, day.*

**Figure 2.**
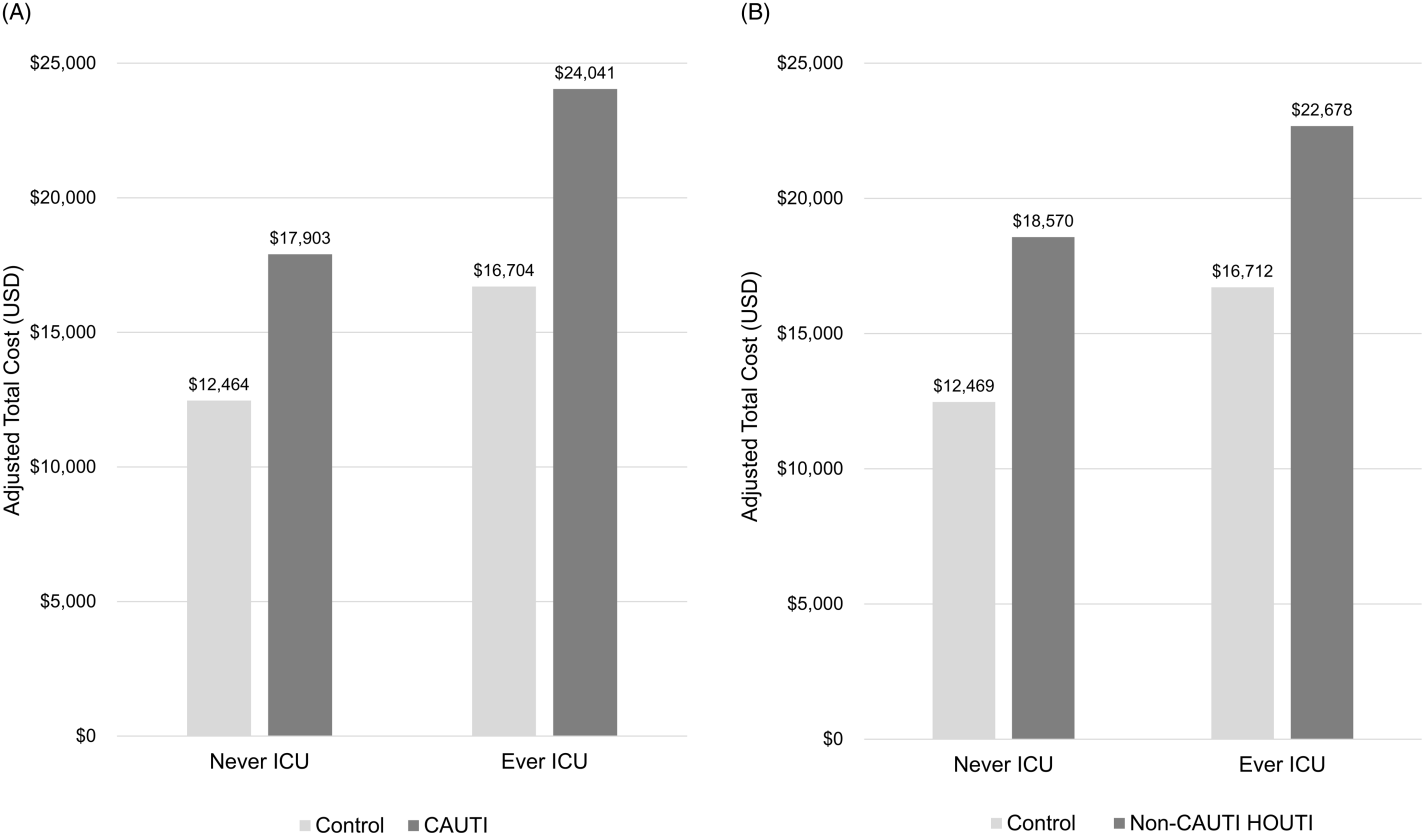
Cost of CAUTI and non-CAUTI HOUTI. A. Adjusted total cost of CAUTI (Cohort 2—subjects with LOS ≤ 10 days, and no HOB). B. Adjusted total cost of non-CAUTI HOUTI (Cohort 2—subjects with LOS ≤ 10 days, and no HOB).

**Figure 3.**
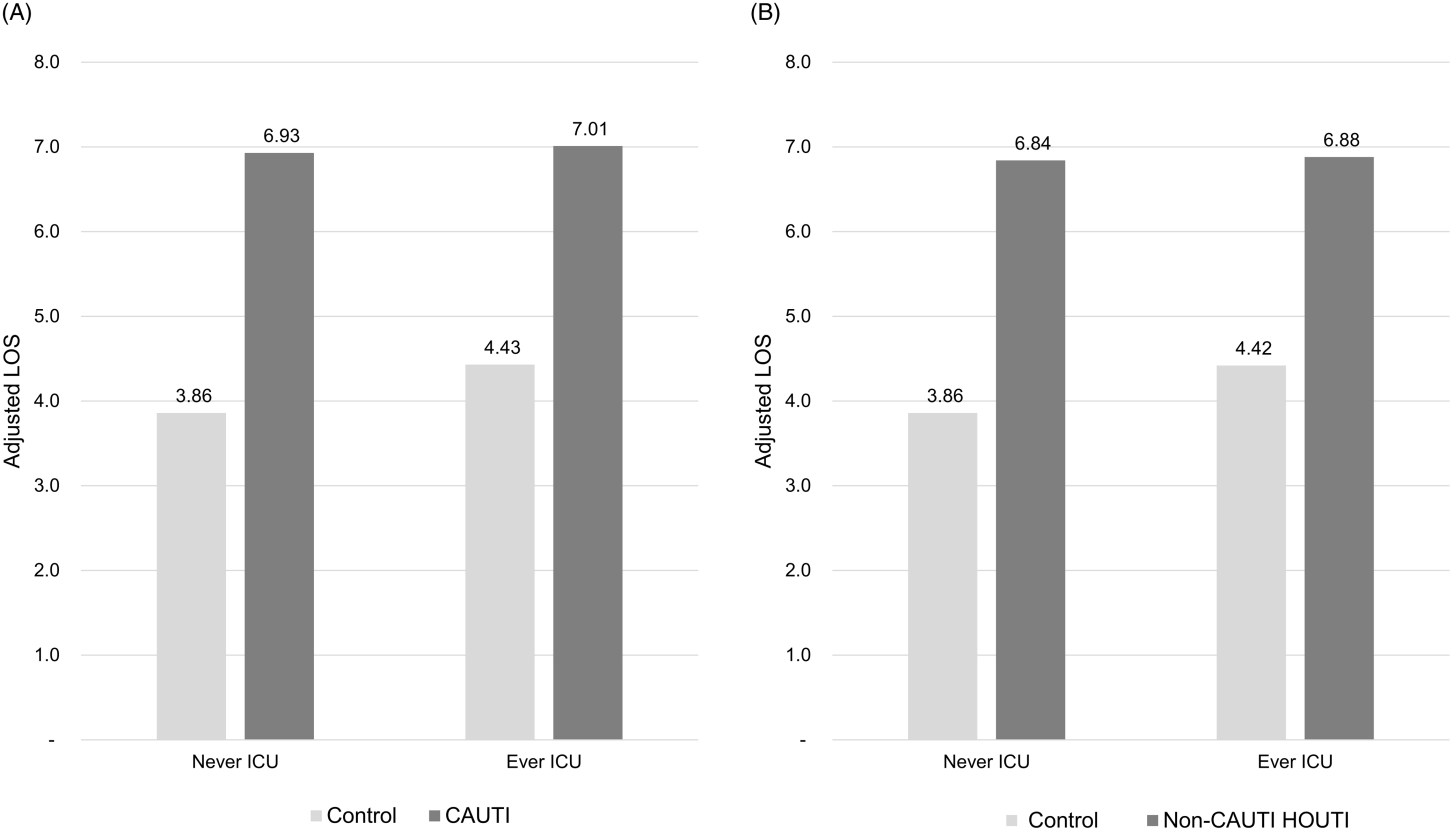
LOS Associated with CAUTI and non-CAUTI HOUTI. A. Adjusted LOS associated with CAUTI (Cohort 2—subjects with LOS ≤ 10 days, and no HOB). B. Adjusted LOS associated with non-CAUTI HOUTI (Cohort 2—subjects with LOS ≤ 10 days, and no HOB).

In the subgroup with LOS ≤10 days and no HOB, incremental adjusted total hospital cost was significantly higher for CAUTI patients: $5,439 (*P* < .01) with no ICU stay and $7,337 (*P* < .0001) with an ICU stay (Figure 2A). Similarly, non-CAUTI HOUTI patients had higher incremental adjusted total hospital costs: $6,101 (*P* < .0001) with no ICU stay and $5,966 (*P* < .0001) with an ICU stay (Figure 2B). Neither CAUTI nor non-CAUTI HOUTI had a significant impact on either 30-day readmission rates or mortality with the exception of CAUTI patients who had been in the ICU exhibiting a 2.14X greater risk of mortality than matched controls (*P* < .05, Table 3). Incremental adjusted LOS was significantly longer for CAUTI patients: 3.07 days (*P* < .0001) with no ICU stay and 2.58 days (*P* < .0001) with an ICU stay (Figure 3A). Similarly, non-CAUTI HOUTI patients had longer incremental adjusted LOS: 2.98 days (*P* < .0001) with no ICU stay and 2.46 days (*P* < .0001) with an ICU stay (Figure 3B).

## Discussion

In the general hospital population of adults, the proportion of non-CAUTI HOUTI was over 7-fold greater compared to CAUTI. The attributable risk of increased LOS and total hospital cost of both CAUTI and non-CAUTI HOUTI were significantly higher compared to controls. Though the risk of likely secondary HOB was higher in patients with CAUTI vs non-CAUTI HOUTI, the volume of HOB was three times greater in non-CAUTI HOUTI. This finding has implications for infection control programs as non-CLABSI HOB has been shown to sustain even higher incremental risk for longer LOS, higher hospital costs, and depending on ICU status, higher mortality and 30-day readmission risk.^12^

These findings highlight the importance of recognizing both CAUTI and non-CAUTI HOUTI and are complemented by a recent practice recommendation to establish a system for defining, analyzing, and reporting data on non-catheter-associated UTIs.^21^ While CAUTI remains the more costly of the two infections, this analysis suggests that reducing not only CAUTI, but also non-CAUTI HOUTI, may result in improved patient outcomes and lower overall HOB rates. *E. coli* bloodstream infection rate is a UK National Health Service oversight metric^22^ and research in the UK has found that *E. coli* bacteremia is most frequently caused by CAUTI.^23^ This is worthy of attention given that the Centers for Medicare & Medicaid Services is currently reviewing an all-cause HOB metric.^24^

As the definition for CAUTI has become more stringent, the prevalence of CAUTI has declined.^7,8,10^ This study found that only 12.0% of all HOUTIs were identified as CAUTIs and thus the vast majority of HOUTIs did not meet currently reportable NHSN criteria. An analysis of cases of hospital-onset BSI, secondary to HOUTI, following introduction of the 2015 NHSN CAUTI definition changes, found that 41.9% of those HOB infections could not be classified as secondary to CAUTI because they were either fungal in nature or because the bacterial urine culture was <100,000 CFU/mL.^25^ Our analysis builds upon these data, highlights the importance of both types of HOUTI and associated patient outcomes, and suggests a clinical opportunity for infection prevention efforts to focus on the more serious clinical outcomes of HOB via all-cause HOUTIs irrespective of indwelling urinary catheter use or limitations of pathogen levels and species.

Prior analyses of the cost of CAUTI have yielded low values. A 2010 Agency for Healthcare Research and Quality (AHRQ) estimate for the cost of CAUTI was $1,090 increasing more than 10-fold in seven years in a subsequent analysis.^26^ The researchers noted that efforts to reduce the utilization of indwelling urinary catheters, and CAUTI definition changes, likely concentrated the remaining CAUTI cases to those patients with severe, more costly infections. Systematic reviews have previously estimated the cost of CAUTI to range from $1,768 to $10,197 (Medicare beneficiaries without and with ICU stays, 2016 USD)^27^ to $13,793 (2015 USD).^26^ This large range may be partially explained by the acuity associated with an extended LOS. Stratification by LOS and HOB during the hospitalization showed that the attributable cost of CAUTI was as low as $5,439 to $7,337 and was responsible for 2.58 to 3.07 additional LOS days in patients with LOS of ≤10 days and no matched HOB, depending on ICU status during the hospitalization.

This study is one of the first to quantify the attributable cost of non-CAUTI HOUTI. The attributable cost of non-CAUTI HOUTI was approximately $6,000 contributing 2.46 to 2.98 additional LOS days, depending on ICU status, in the least acute group of patients analyzed. This value is plausible and consistent with prior studies.^28^ It is within the lower end of the 95% CI range for CAUTI ($5,019 to $22,568, 2015 USD) set forth in the AHRQ report^26^ and similar to the $6,424 (2011 USD) cost of a UTI admission determined by an analysis of the Nationwide Inpatient Sample,^29^ both of which were published in 2017. We therefore augment these analyses with more recent outcomes and cost data.

This study had several limitations. The analyzed hospitals were limited to those where total cost of care per admission and NHSN HAI reporting were both readily available. The urine culture stewardship practices of the individual hospitals were unknown, for example, it is possible that cultures may have been ordered less frequently for patients with an indwelling catheter. The association between potential secondary HOB and HOUTI may have been underestimated due to the following: (1) application of a stringent definition of HOB which required it to occur on day 4 or later and within 48 hours of the first positive urine culture collection date, and (2) patients with admissions for infection were excluded potentially eliminating the highest acuity patients with HOUTI. The location of the urine specimen collection may not have been the true location of infection onset depending on patient movement between infection onset and when the specimen was obtained. In addition, with no access to notes in the medical record, the mechanism for collecting the urine specimen and/or the mechanism of urine management, including the use of indwelling urinary catheters, could not be ascertained (note that uropathogen growth ≥10,000 CFU/mL is considered significant in specimens obtained with an in-and-out catheter per ASM recommendations in the Clinical Microbiology Procedures Handbook^16^), additional LOS could not be ascribed to the HOUTI with absolute certainty. Accounting for potential confounding was done at the design and analysis phase of the study. However, residual confounding may still be possible as with all observational studies. Finally, as the CDC definition of HOB is still evolving, the definition employed in this analysis is a signal of HOB burden and not the exact definition that may ultimately be used by the CDC and NHSN.

Despite these limitations, the study had several strengths. A stringent definition for non-CAUTI HOUTI required cases to have both: (1) an algorithmically derived indication of a likely hospital-onset UTI based on the positive urine culture, and (2) an order for complementary antimicrobials within 48 hours of that first positive culture (resulting in exclusion of 32.8% of HOUTIs to arrive at our cohort of 3,177 cases of non-CAUTI HOUTI). It is thus highly unlikely that asymptomatic bacteriuria was captured in any of the analyses. In addition, a large sample of US hospitals was utilized including sites with and without academic affiliations. Lastly, the database included both laboratory results and pharmacy orders providing a novel opportunity to evaluate non-CAUTI HOUTI and its association with HOB.

In conclusion, non-CAUTI HOUTI is a common and potentially preventable source for HOB that is associated with longer LOS and higher hospital costs compared to those in similar patients without an infection. While a higher percentage of CAUTI cases evolve into HOB, non-CAUTI HOUTI-related HOB occur more often. These data support the ongoing surveillance of not only CAUTI but also non-CAUTI HOUTI as the latter may also represent a vulnerable population that could benefit from surveillance and prevention efforts, particularly in the non-ICU setting.

## Supporting information

Kelly et al. supplementary material Kelly et al. supplementary material

## Supplementary material

The supplementary material for this article can be found at https://doi.org/10.1017/ice.2024.26.

## References

[1] Klevens RM , Edwards JR , Richards CL, Jr., et al. Estimating health care-associated infections and deaths in U.S. hospitals, 2002. Public Health Rep 2007;122:160–6. doi: 10.1177/003335490712200205 17357358 PMC1820440

[2] Magill SS , O’Leary E , Janelle SJ , et al. Changes in prevalence of health care-associated infections in U.S. hospitals. N Engl J Med 2018;379:1732–1744. doi: 10.1056/NEJMoa1801550 30380384 PMC7978499

[3] Umscheid CA , Mitchell MD , Doshi JA , Agarwal R , Williams K , Brennan PJ. Estimating the proportion of healthcare-associated infections that are reasonably preventable and the related mortality and costs. Infect Control Hosp Epidemiol 2011;32:101–14. doi: 10.1086/657912 21460463

[4] Gould CV , Umscheid CA , Agarwal RK , Kuntz G , Pegues DA. Guideline for prevention of catheter-associated urinary tract infections 2009. Infect Control Hosp Epidemiol 2010;31:319–26. doi: 10.1086/651091 20156062

[5] Dudeck MA , Horan TC , Peterson KD , et al. National Healthcare Safety Network (NHSN) report, data summary for 2009, device-associated module. Am J Infect Control 2011;39:349–67. doi: 10.1016/j.ajic.2011.04.011 21774120

[6] National Healthcare Safety Network (NHSN). Paving the path forward: 2015 rebaseline. FAQs: NHSN CAUTI Definition & Rebaseline, last reviewed June 14, 2023. https://www.cdc.gov/nhsn/pdfs/rebaseline/faq-cauti-rebaseline.pdf. Published 2015. Accessed January 3, 2024.

[7] Sopirala MM , Syed A , Jandarov R , Lewis M. Impact of a change in surveillance definition on performance assessment of a catheter-associated urinary tract infection prevention program at a tertiary care medical center. Am J Infect Control 2018;46:743–746. doi: 10.1016/j.ajic.2018.01.019 29551201

[8] Press MJ , Metlay JP. Catheter-associated urinary tract infection: does changing the definition change quality? Infect Control Hosp Epidemiol 2013;34:313–5. doi: 10.1086/669525 23388369 PMC3573527

[9] Centers for Disease Control and Prevention. Urinary tract infection (catheter-associated urinary tract infection [CAUTI] and non-catheter-associated urinary tract infection [UTI]) events. National Healthcare Safety Network, 2024. https://www.cdc.gov/nhsn/pdfs/pscmanual/7psccauticurrent.pdf. Published 2024. Accessed January 3, 2024.

[10] Fakih MG , Groves C , Bufalino A , Sturm LK , Hendrich AL. Definitional change in NHSN CAUTI was associated with an increase in CLABSI events: evaluation of a large health system. Infect Control Hosp Epidemiol 2017;38:685–689. doi: 10.1017/ice.2017.41 28330520

[11] Yu KC , Ye G , Edwards JR , et al. Hospital-onset bacteremia and fungemia: an evaluation of predictors and feasibility of benchmarking comparing two risk-adjusted models among 267 hospitals. Infect Control Hosp Epidemiol 2022;43:1317–1325. doi: 10.1017/ice.2022.211 36082774 PMC9588439

[12] Yu KC , Jung M , Ai C . Characteristics, costs, and outcomes associated with central-line-associated bloodstream infection and hospital-onset bacteremia and fungemia in US hospitals. Infect Control Hosp Epidemiol 2023:1–7. doi: 10.1017/ice.2023.132 PMC10755163 37424226

[13] Yu KC , Ye G , Edwards JR , et al. Treated, hospital-onset Clostridiodes difficile infection: An evaluation of predictors and feasibility of benchmarking comparing 2 risk-adjusted models among 265 hospitals. Infect Control Hosp Epidemiol 2023:1–9. doi: 10.1017/ice.2023.124 PMC10782205 37449415

[14] Brossette SE , Hacek DM , Gavin PJ , et al. A laboratory-based, hospital-wide, electronic marker for nosocomial infection: the future of infection control surveillance? Am J Clin Pathol 2006;125:34–9. 16482989

[15] Ridgway JP , Sun X , Tabak YP , Johannes RS , Robicsek A. Performance characteristics and associated outcomes for an automated surveillance tool for bloodstream infection. Am J Infect Control 2016;44:567–71. doi: 10.1016/j.ajic.2015.12.044 26899530

[16] Yarbrough ML , Potter RF . 3.11.1 Urine cultures. In: Clinical Microbiology Procedures Handbook, 5th edition. Washington, DC: ASM Press; 2023.

[17] Centers for Disease Control and Prevention. Hospital toolkit for adult sepsis surveillance, 2018. https://www.cdc.gov/sepsis/pdfs/sepsis-surveillance-toolkit-mar-2018_508.pdf. Published 2018. Accessed January 3, 2024.

[18] National Healthcare Safety Network. National Healthcare Safety Network (NHSN) patient safety component manual, 2023. https://www.cdc.gov/nhsn/pdfs/pscmanual/pcsmanual_current.pdf. Published 2024. Accessed August 10, 2023.

[19] Tabak YP , Sun X , Nunez CM , Johannes RS. Using electronic health record data to develop inpatient mortality predictive model: Acute Laboratory Risk of Mortality Score (ALaRMS). J Am Med Inform Assoc 2014;21:455–63. doi: 10.1136/amiajnl-2013-001790 24097807 PMC3994855

[20] Halpern NA , Pastores SM. Critical care medicine in the United States 2000-2005: an analysis of bed numbers, occupancy rates, payer mix, and costs. Crit Care Med 2010;38:65–71. doi: 10.1097/CCM.0b013e3181b090d0 19730257

[21] Patel PK , Advani SD , Kofman AD , et al. Strategies to prevent catheter-associated urinary tract infections in acute-care hospitals: 2022 update. Infect Control Hosp Epidemiol 2023;44:1209–1231. doi: 10.1017/ice.2023.137 37620117 PMC11287483

[22] National Health Service England. NHS oversight metrics for 2022/23 National Health Service England, 2022. https://www.england.nhs.uk/publication/nhs-oversight-framework-22-23/. Published 2022. Accessed January 3, 2024.

[23] Melzer M , Wickramasinghe D , Welch C. Outcomes in consecutive hospitalized UK patients with bacteraemia or fungaemia caused by medical devices and procedures. J Hosp Infect 2015;91:146–52. doi: 10.1016/j.jhin.2015.06.015 26275709

[24] Stack MA , Dbeibo L , Fadel W , Kelley K , Sadowski J , Beeler C. Etiology and utility of hospital-onset bacteremia as a safety metric for targeted harm reduction. Am J Infect Control 2024;52:195–199. doi: 10.1016/j.ajic.2023.06.002 37295676

[25] Greene MT , Ratz D , Meddings J , Fakih MG , Saint S. Potential misclassification of urinary tract-related bacteremia upon applying the 2015 catheter-associated urinary tract infection surveillance definition from the National Healthcare Safety Network. Infect Control Hosp Epidemiol 2016;37:469–71. doi: 10.1017/ice.2015.339 26778287 PMC5176094

[26] Bysshe T , Gao Y , Heaney-Huls K , et al. Estimating the Additional Hospital Inpatient Cost and Mortality Associated with Selected Hospital-Acquired Conditions. Rockville, MD: Agency for Healthcare Research and Quality. AHRQ Publication No. 18-0011-EF; 2017.

[27] Hollenbeak CS , Schilling AL. The attributable cost of catheter-associated urinary tract infections in the United States: a systematic review. Am J Infect Control 2018;46:751–757. doi: 10.1016/j.ajic.2018.01.015 29478760

[28] Tabak YP , Sung AH , Ye G , Vankeepuram L , Gupta V , McCann E. Attributable clinical and economic burden of carbapenem-non-susceptible Gram-negative infections in patients hospitalized with complicated urinary tract infections. J Hosp Infect 2019;102:37–44. doi: 10.1016/j.jhin.2018.11.018 30503367

[29] Simmering JE , Tang F , Cavanaugh JE , Polgreen LA , Polgreen PM. The increase in hospitalizations for urinary tract infections and the associated costs in the United States, 1998-2011. Open Forum Infect Dis 2017;4:ofw281. doi: 10.1093/ofid/ofw281 28480273 PMC5414046

