# Supplementary material for: Catheter-associated urinary tract infections (CAUTIs) and non-CAUTI hospital-onset urinary tract infections: Relative burden, cost, outcomes and related hospital-onset bacteremia and fungemia infections
Source: Infect Control Hosp Epidemiol. 2024 Feb 20;45(7):864–71. doi: 10.1017/ice.2024.26 (PMC11439594; doi:10.1017/ice.2024.26)
Supplement: Kelly et al. supplementary material [file S0899823X24000266sup001.docx]

**SUPPLEMENTAL DATA**

## **Supplemental Table 1. Study Attrition.**

| **Inclusion/Exclusion criteria** | **Hospitals N** | **Subject Cohort N** | **Exclude N** | **Exclude %** |
| --- | --- | --- | --- | --- |
| Total hospitals \| Acute care admissions | 85 | 1,149,448 |  |  |
| Include hospitals with at least 1 CAUTI and reporting of CAUTI to NHSN | 43 | 674,611 | 474,837 | 41% |
| Include subjects if 2 day < LOS < 365 days with patient age ≥ 18 years, and admission is not for infection DRG | 43 | 549,433 (Cohort 1) | 125,178 | 11% |
| Exclude control with potential infection* | 43 | 427,005 | 122,428 | 11% |
| Exclude case without antimicrobial use for clinical approval | 43 | 425,423 | 1,582 | 0% |
| Exclude if missing ICU status | 43 | 420,368 | 6,637 | 1% |
| Exclude if age is outside of cases | 43 | 420,368 | 0 | 0% |
| Exclude if MDC does not match case | 43 | 347,774 | 72,594 | 6% |
| Exclude if DRG does not match case | 43 | 160,666 | 187,108 | 16% |
| Exclude if primary procedure coding system does not match case | 40 | 82,787 (Cohort 2) | 264,987 | 7% |

**Use of antibiotics >72 hours. Note: Cohort 2 was comprised of 82,787 subjects [360 (0.4%) with CAUTI; 1,513 (1.8%) with non-CAUTI HOUTI; and 80,914 controls (97.7%)].*

**Supplemental Table 2. Urine Culture and Antimicrobial Susceptibility Testing (AST) Analysis by Pathogen Type**

|  | **Bacteria and/or Fungi** | **Only Fungi Isolate** | **No Identified Pathogen** |
| --- | --- | --- | --- |
| **CAUTI***  **N=434** |  |  |  |
| ≥100,000 CFU/ml with AST | 405 (93.3%) | - | - |
| >10,000 and <100,000 CFU/ml with AST | 0 (0.0%) | - | - |
| ≥100,000 CFU/ml with no AST | 12 (2.8%) | - | - |
| >10,000 and <100,000 CFU/ml with no AST | 1 (0.2%) | - | - |
| No identified pathogen | - | - | 16 (3.7%) |
| **Non-CAUTI HOUTI**  **N=3,177** |  |  |  |
| ≥100,000 CFU/ml with AST | 2,292 (72.1%) | 108 (3.4%) | - |
| >10,000 and <100,000 CFU/ml with AST | 528 (16.6%) | 2 (0.1%) | - |
| ≥100,000 CFU/ml with no AST | 126 (4.0%) | 0 (0.0%) | - |
| >10,000 and <100,000 CFU/ml with no AST | 68 (2.1%) | 53 (1.7%) | - |

All infections had a new antimicrobial order lasting greater than 24 hours ordered ±2 days of the specimen collection date. For the fungi infections, 161 were treated with fluconazole, 1 was treated with voriconazole and 1 was treated with both antifungals. *Note: *confirmed by Hospital Infection Preventionist.*

## **Supplemental Table 3. Patient Demographic Distribution by CAUTI and non-CAUTI HOUTI Status (Cohort 2).**

|  | **Overall** | **CAUTI** | **Non-CAUTI HOUTI** | **Control** |
| --- | --- | --- | --- | --- |
|  | **N=82,787** | **N=360** | **N=1,513** | **N=80,914** |
| **Patient Characteristics** |  |  |  |  |
| Age group (years) |  |  |  |  |
| 18-40 | 5,640 (6.8%) | 30 (8.3%) | 80 (5.3%) | 5530 (6.8%) |
| 41-64 | 29,814 (36.0%) | 129 (35.8%) | 459 (30.3%) | 29,226 (36.1%) |
| 65-80 | 33,271 (40.2%) | 146 (40.6%) | 645 (42.6%) | 32,480 (40.1%) |
| >80 | 14,062 (17.0%) | 55 (15.3%) | 329 (21.7%) | 13,678 (16.9%) |
| Age |  |  |  |  |
| Mean (SD) | 65.6 (15.0) | 64.9 (15.4) | 68.3 (14.6) | 65.6 (15.0) |
| Median [Q1, Q3] | 67.0 [57.0, 77.0] | 67.0 [57.0, 75.0] | 70.0 [60.0, 79.0] | 67.0 [57.0, 77.0] |
| Female | 43,579 (52.6%) | 191 (53.1%) | 971 (64.2%) | 42,417 (52.4%) |
| ALaRMS Score^19^, mean (SD) | 46.5 (18.9) | 56.8 (21.6) | 56.4 (19.8) | 46.2 (18.8) |
| Payor |  |  |  |  |
| Medicaid | 5581 (6.7%) | 47 (13.1%) | 148 (9.8%) | 5,386 (6.7%) |
| Medicare | 55,246 (66.7%) | 243 (67.5%) | 1,092 (72.2%) | 53,911 (66.6%) |
| Other | 1702 (2.1%) | 10 (2.8%) | 29 (1.9%) | 1,663 (2.1%) |
| Private | 17,842 (21.6%) | 52 (14.4%) | 195 (12.9%) | 17,595 (21.7%) |
| Uninsured | 2298 (2.8%) | 7 (1.9%) | 48 (3.2%) | 2,243 (2.8%) |
| **Hospital Characteristics** |  |  |  |  |
| Staffed bed size |  |  |  |  |
| <100 | 3,110 (3.8%) | 5 (1.4%) | 41 (2.7%) | 3,064 (3.8%) |
| 100-300 | 22,903 (27.7%) | 64 (17.8%) | 365 (24.1%) | 22,474 (27.8%) |
| >300 | 56,774 (68.6%) | 291 (80.8%) | 1,107 (73.2%) | 55,376 (68.4%) |
| Teaching hospital status | 51,668 (62.4%) | 265 (73.6%) | 1,081 (71.4%) | 50,322 (62.2%) |
| Urban | 66,147 (79.9%) | 299 (83.1%) | 1,260 (83.3%) | 64,588 (79.8%) |

## **ALaRMS scores for Cohorts 1 and 2 were comparable.*

## **Supplemental Table 4. Association Between CAUTI and Non-CAUTI HOUTI with Outcomes in Patients with LOS > 10 days and No Secondary HOB.**

|  | **Never-ICU** | | |  | **Ever-ICU** | | |
| --- | --- | --- | --- | --- | --- | --- | --- |
|  | **CAUTI**  **N=39** | **Control**  **N=2,334** | **Difference/ RR** |  | **CAUTI,**  **N=203** | **Control**  **N=2,849** | **Difference/ RR** |
| **30-day Readmission** | | | | | | | |
| Unadjusted | 2 (5.1%) | 47 (2.0%) |  |  | 20 (9.9%) | 437 (15.3%) |  |
| Adjusted | - | - | 0.95^ns^ |  | 0.06 (0.03, 0.11) | 0.11 (0.08, 0.15) | 0.57* |
| **Mortality** | | | | | | | |
| Unadjusted | 0 (0%) | 621 (1.1%) |  |  | 29 (14.3%) | 157 (5.5%) |  |
| Adjusted | - | - | - |  | 0.09 (0.05, 0.17) | 0.04 (0.02, 0.06) | 2.66** |
| **Length of Stay** | | | | | | | |
| Unadjusted | 17.0 [13.0, 27.0] | 13.0 [12.0, 16.0] |  |  | 24.0 [16.0, 37.5] | 13.0 [12.0, 16.0] |  |
| Adjusted | 23.19  (21.47, 25.04) | 14.49  (13.88, 15.12) | 8.70*** |  | 30.27  (28.55, 32.09) | 14.53  (13.78, 15.32) | 15.74*** |
| **Total Hospital Cost** | | | | | | | |
| Unadjusted | 34,000  [22,200, 58,300] | 24,000  [18,400, 32,500] |  |  | 65,600  [43,600, 112,000] | 37,800  [27,500, 54,200] |  |
| Adjusted | 52,397  (42,750, 64,220) | 28,893  (24,494, 34,082) | 23,504*** |  | 94,471  (77,213, 115,585) | 42,957  (35,472, 52,022) | 51,513*** |
|  | **Non-CAUTI HOUTI**  **N=303** | **Control**  **N=2,334** | **Difference/ RR** |  | **Non-CAUTI HOUTI**  **N=742** | **Control**  **N=2,849** | **Difference/ RR** |
| **30-day Readmission** | | | | | | | |
| Unadjusted | 58 (19.1%) | 439 (18.8%) |  |  | 113 (15.2%) | 437 (15.3%) |  |
| Adjusted | 0.17 (0.11, 0.24) | 0.16 (0.12, 0.22) | 1.01 |  | 0.11 (0.08, 0.15) | 0.11 (0.08, 0.16) | 0.94 |
| **Mortality** | | | | | | | |
| Unadjusted | 14 (4.6%) | 47 (2.0%) |  |  | 108 (14.6%) | 157 (5.5%) |  |
| Adjusted | 0.03 (0.01, 0.1) | 0.02 (0.01, 0.05) | 1.97* |  | 0.12 (0.07, 0.18) | 0.04 (0.03, 0.07) | 2.69*** |
| **Length of Stay** | | | | | | | |
| Unadjusted | 16.0 [12.5, 23.0] | 13.0 [12.0, 16.0] |  |  | 21.0 [15.0, 30.0] | 13.0 [12.0, 16.0] |  |
| Adjusted | 20.57 (19.57, 21.61) | 14.61 (13.98, 15.26) | 5.96*** |  | 24.31  (23.17, 25.49) | 14.24  (13.6, 14.92) | 10.06*** |
| **Total Hospital Cost** | | | | | | | |
| Unadjusted | 29,200  [21,800, 47,500] | 24,000  [18,400, 32,500] |  |  | 56,300 [37,900, 89,100] | 37,800 [27,500, 54,200] |  |
| Adjusted | 44,448  (37,513, 52,664) | 30,049  (25,511, 35,394) | 14,399*** |  | 71265 (58,857, 86,290) | 42,626 (35,286, 51,494) | 28,639*** |

*P-value: ^ns^ not statistically significant; *<0.05; **<0.01; ***<0.0001. Models were adjusted for age, sex, ALaRMS score, and hospital-level variables (payer, staffed bed size, teaching status, and urbanicity).*

## **Supplemental Table 5. Association Between CAUTI and Non-CAUTI HOUTI with Outcomes in Patients with LOS > 10 days and Secondary HOB.**

|  | **Never-ICU** | | |  | **Ever-ICU** | | |
| --- | --- | --- | --- | --- | --- | --- | --- |
|  | **CAUTI**  **N=7** | **Control**  **N=2,334** | **Difference/ RR** |  | **CAUTI**  **N=38** | **Control**  **N=2,849** | **Difference/ RR** |
| **30-day Readmission** | | | | | | | |
| Unadjusted | 3 (42.9%) | 439 (18.8%) |  |  | 1 (2.6%) | 437 (15.3%) |  |
| Adjusted | 0.36 (0.11, 0.73) | 0.16 (0.12, 0.22) | 2.22 |  | 0.02 (0, 0.12) | 0.1 (0.07, 0.14) | 0.17 |
| **Mortality** | | | | | | | |
| Unadjusted | 0 (0%) | 47 (2.0%) |  |  | 9 (23.7%) | 157 (5.5%) |  |
| Adjusted | - | - | - |  | 0.17 (0.07, 0.36) | 0.03 (0.02, 0.05) | 5.76*** |
| **Length of Stay** | | | | | | | |
| Unadjusted | 21.0 [15.0, 23.5] | 13.0 [12.0, 16.0] |  |  | 22.5 [17.0, 30.8] | 13.0 [12.0, 16.0] |  |
| Adjusted | 20.66 (17.46, 24.44) | 14.68 (14.07, 15.33) | 5.97*** |  | 28.94 (26.93, 31.1) | 14.61 (14.03, 15.2) | 14.33*** |
| **Total Hospital Cost** | | | | | | | |
| Unadjusted | 43,700  [33,900, 56,900] | 24,000  [18,400, 32,500] |  |  | 85,200 [52,200, 117,000] | 37,800  [27,500, 54,200] |  |
| Adjusted | 59,947  (43,436, 82,735) | 29,675  (25,206, 34,936) | 30,272*** |  | 100,022  (78,724, 127,084) | 42,437  (35,060, 51,365) | 57,585*** |
|  | **Non-CAUTI HOUTI**  **N=19** | **Control**  **N=2,334** | **Difference/ RR** |  | **Non-CAUTI HOUTI**  **N=60** | **Control**  **N=2,849** | **Difference/ RR** |
| **30-day Readmission** | | | | | | | |
| Unadjusted | 2 (10.5%) | 439 (18.8%) |  |  | 5 (8.3%) | 437 (15.3%) |  |
| Adjusted | 0.09 (0.02, 0.3) | 0.16 (0.12, 0.22) | 0.52 |  | 0.05 (0.02, 0.13) | 0.1 (0.07, 0.15) | 0.510 |
| **Mortality** | | | | | | | |
| Unadjusted | 2 (10.5%) | 47 (2.0%) |  |  | 13 (21.7%) | 157 (5.5%) |  |
| Adjusted | - | - | - |  | 0.13 (0.06, 0.27) | 0.03 (0.02, 0.05) | 4.49*** |
| **Length of Stay** | | | | | | | |
| Unadjusted | 23.0 [17.5, 27.5] | 13.0 [12.0, 16.0] |  |  | 23.0 [16.0, 34.0] | 13.0 [12.0, 16.0] |  |
| Adjusted | 30.36 (27.62, 33.37) | 14.79 (14.14, 15.47) | 15.57*** |  | 26.84  (25.22, 28.57) | 14.46  (13.89, 15.06) | 12.38*** |
| **Total Hospital Cost** | | | | | | | |
| Unadjusted | 47,000 [25,600, 81,900] | 24,000 [18,400, 32,500] |  |  | 62,100 [40,500, 105,000] | 37,800 [27,500, 54,200] |  |
| Adjusted | 60,135  (47,508, 76,119) | 29,771 (25,292, 35,044) | 30,364*** |  | 78,580  (62,943, 98,102) | 42,567  (35,194, 51,483) | 36,013*** |

*P-value: ^ns^ not statistically significant; *<0.05; **<0.01; ***<0.0001. Models were adjusted for age, sex, ALaRMS score, and hospital-level variables (payer, staffed bed size, teaching status, and urbanicity)*
